# Detection of Orientia *sp.* DNA in rodents from Asia, West Africa and Europe

**DOI:** 10.1186/s13071-015-0784-7

**Published:** 2015-03-21

**Authors:** Jean François Cosson, Maxime Galan, Emilie Bard, Maria Razzauti, Maria Bernard, Serge Morand, Carine Brouat, Ambroise Dalecky, Khalilou Bâ, Nathalie Charbonnel, Muriel Vayssier-Taussat

**Affiliations:** INRA, CBGP, 755 Avenue Campus Agropolis, Montferrier sur Lez, CS30016, 34988 France; INRA, EpiA, Clermont-Ferrand, France; INRA, GABI, Sigenae, Domaine de Vilvert, Jouy en Josas, 78352 France; CNRS-CIRAD, Centre d’Infectiologie Christophe Mérieux du Laos, Vientiane, Lao PDR; Department of Helminthology, Faculty of Tropical Medicine, Mahidol University, Bangkok, Thailand; Ird, CBGP, 755 avenue du campus Agropolis, Montferrier sur Lez cedex, CS 30016, 34988 France; INRA, Bipar, 23 Av. Général de Gaulle, Maisons-Alfort, France; IRD, Aix Marseille Université, LPED (UMR IRD-AMU), Marseille, France; Ird, CBGP, Campus ISRA/IRD de Bel Air, BP 1386, Dakar, CP 18524 Senegal

**Keywords:** Scrub typhus, Zoonoses, Emerging disease, Rodent-borne disease, Metagenomics

## Abstract

*Orientia* bacterium is the agent of the scrub typhus, a seriously neglected life-threatening disease in Asia. Here, we report the detection of DNA of *Orientia* in rodents from Europe and Africa. These findings have important implications for public health. Surveillance outside Asia, where the disease is not expected by sanitary services, needs to be improved.

## Findings

*Orientia tsutsugamushi* is the only known species belonging to the *Orientia* bacterial genus. The bacterium causes scrub typhus in humans. It is an obligate intracytosolic bacterium that is transmitted during feeding by larval trombiculid mites, and is hosted by rodents [1]. In Asia, approximately one million cases of scrub typhus occur annually, where it is probably one of the most underdiagnosed and underreported febrile illnesses requiring hospitalization [2], with an estimated 10% fatality rate unless treated appropriately. Formerly thought to be geographically restricted to Asia [3], *Orientia* was recently identified in sick patients from the Arabian Peninsula [4] and Chile [5]. Miscellaneous reports of scrub typhus-like illness have previously questioned the presence of the bacterium in the Congo [6] and Cameroon [7].

## Methods

In order to generate a global picture of zoonotic bacteria that are likely to be harboured by rodents, we applied a metagenomic approach using spleen samples of 1334 rodents from France (Ardennes region), Senegal (along the Senegal River) and Thailand (northern and north-eastern provinces of Loei, Nan and Buriram). Rodents were trapped in both natural habitats and villages within rural landscapes. They were euthanized by cervical dislocation and dissected. In order to prevent cross contamination during dissection, we systematically alternated the use of two sets of dissecting instruments. After dissecting a rodent, the set used was immersed in bleach then water and let in alcohol, while we dissected another rodent with the other set [8]. Spleens were placed in RNA*later*® storage solution (Sigma-Aldrich, Saint Louis, MO, USA) then stored at -20°C until further analysis. Genomic DNA was then extracted from the spleen using the DNeasy® 96 Tissue Kit (Qiagen, Germany). Spleen DNA samples were screened for the presence of bacteria using universal primers targeting the hyper variable region V4 of the *16S rRNA* gene (251 bp) via Illumina MiSeq (Illumina) sequencing. The V4 region has been proven to have excellent taxonomic resolution at the genus level [9]. A multiplexing strategy enabled the identification of bacterial genera in each individual sample. We followed the method described in [10] to perform PCR amplification, indexing, pooling, multiplexing and de-multiplexing and finally taxonomic identification using the SILVA SSU Ref NR 119 database as a reference (http://www.arb-silva.de/projects/ssu-ref-nr/). In total we performed four different MiSeq runs, two with rodents from France (N = 557), one with rodents from Asia (N = 423) and one with rodents from Africa (N = 354). For each run, we systematically used negative controls (of DNA extraction and PCR) and none were positive for *Orientia*. Though we did not use positive controls for *Orientia* because the bacterium was not expected in European and African samples. We used positive controls for other bacterial genus like *Leptospira*, *Borrelia*, *Bartonella* and *Mycoplasma*, and all were found positive for the expected bacterial genera.

## Results and discussion

From over a total of 1334 rodents tested, 110 were found positive for *Orientia* (Table 1). As expected, *Orientia* was detected in five sampled rodent species from Thailand: *Rattus tanezumi* (5 positives/67 tested), *Rattus exulans* (1/81), *Bandicota savilei* (2/26), *Berylmys bowersi* (1/17), and *Leopoldamys edwardsi* (1/10). More surprisingly, the bacterium was also detected in numerous rodents collected from both France and Senegal. In Senegal, *Orientia* sequences were only identified in the exotic house mouse (*Mus musculus domesticus*) (48 positives/207 tested), while the sympatric endemic multimammate rats (*Mastomys erythroleucus*) were all found to be negative (0/147), suggesting introduction of the bacterium via the exotic rodent. In France, *Orientia* sp. was detected in three rodent species: *Myodes glareolus* (44/302), *Arvicola scherman* (2/64) and *Microtus arvalis* (6/49). Positive for *Orientia* were trapped inside human houses in Asia and Africa, and in close proximity to human dwellings in Asia and France.

**Table 1:** **Numbers of rodent tested and found positive for** ***Orientia*** **sp. for the different rodent species sampled in France, Senegal and Asia**

**Geographic area**	**Rodent species**	**Number tested**	**Number positive**
France	*Myodes glareolus*	302	44
	*Arvicola scherman*	64	2
	*Microtus arvalis*	49	6
	*Microtus agrestis*	7	0
	*Microtus subterraneus*	4	0
	*Apodemus sylvaticus*	67	0
	*Apodemus flavicolis*	34	0
	*Rattus norvegicus*	30	0
Senegal	*Mus musculus*	207	48
	*Mastomys erythroleucus*	147	0
Asia	*Bandicota indica*	20	0
	*Bandicota savilei*	26	2
	*Berylmys berdmorei*	19	0
	*Berylmys bowersi*	17	1
	*Chiropodomys gliroides*	2	0
	*Hapalomys delacouri*	1	0
	*Leopoldamys edwardsi*	10	1
	*Leopoldamys sabanus*	1	0
	*Maxomys surifer*	15	0
	*Menetes berdmorei*	1	0
	*Mus caroli*	14	0
	*Mus cervicolor*	17	0
	*Mus cookii*	11	0
	*Mus fragilicauda*	1	0
	*Niviventer fulvescens*	17	0
	*Rattus argentiventer*	1	0
	*Rattus exulans*	81	1
	*Rattus losea*	32	0
	*Rattus nitidus*	1	0
	*Rattus tanezumi*	67	5
Total		1334	110

In rodents, blood, spleen and other organs are routinely used for *Orientia* PCR detection [11], although one should notice that such assay is limited to the time window of rickettsemia, i.e. when the bacteria are infecting macrophages in peripheral blood. In our experiments we targeted the spleen because this organ is known to act primarily as a blood filter and then appears appropriate for detecting bacteria infecting blood cells. However, although there are many lines of evidence that *Orientia* may chronically infect humans and rodents, the persistence of the bacteria in organs, and spleen in particular, is currently poorly known [12]. Thus we cannot discard the possibility of false negatives in our assays.

**Figure 1:**
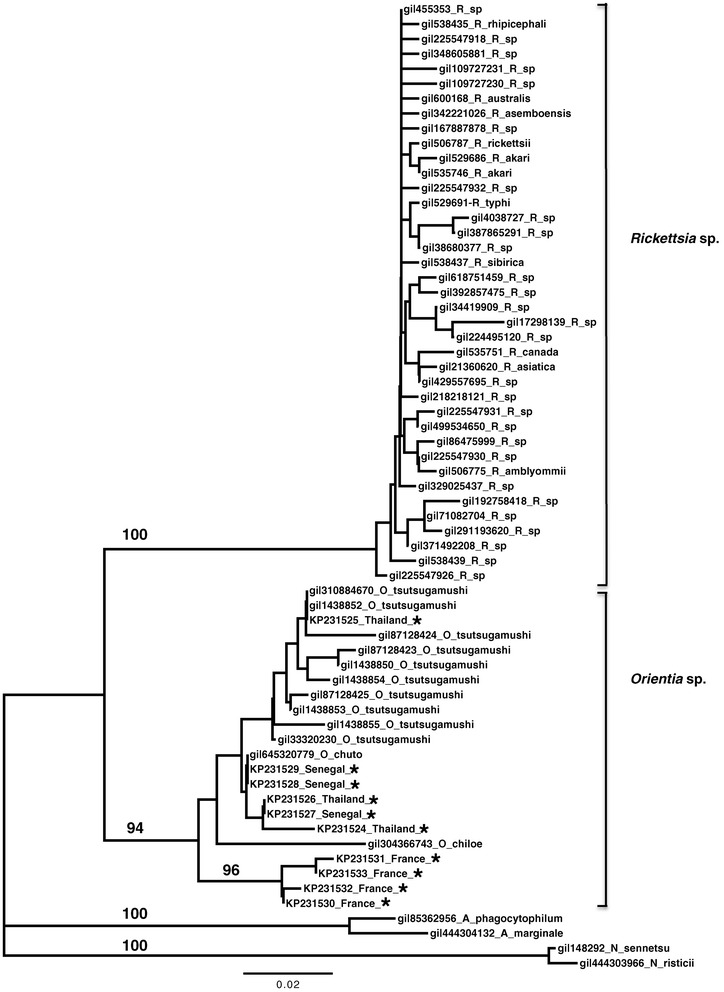
**Phylogenetic tree based on the V4 region of the** ***16S rRNA*** **gene.** GenBank accession numbers are indicated. Only different haplotypes were shown. A complete list of sequences uploaded to GenBank can be provided upon request. Numbers beside branches indicate bootstrap values (>80). O: *Orientia*; R: *Rickettsia*; N: *Neorickettsia*; A: *Anaplasma*. The tree was rooted with the phylogenetically closest genus *Anaplasma* and *Neorickettsia*. Scale bar indicates evolutionary distances. Samples sequenced in the present study are marked with _*.

Sequence analyses revealed that the *Orientia* sequences identified in this study were between 100 to 94.4% identical to GenBank-published *Orientia* sequences isolated from humans, mites and rodents in Asia. All sequences shared only 90.8 to 86.5% identity with GenBank-published *Rickettsia*, the closest bacterial genus to *Orientia*, thus consolidating our finding on the presence of *Orientia* in Europe and Africa. We also performed phylogenetic analyses of both the haplotypes identified in this study, and those from GenBank databases, using the neighbor-joining method [13]. Bootstrap analysis was performed on 1,000 replicates. Haplotypes from this study clustered strongly within the *Orientia* phylogroup and were clearly separated from the *Rickettsia* phylogroup. Asian haplotypes were distributed amongst Genbank haplotypes from Asia and Arabian peninsula (Chuto haplotype), African haplotypes fell with the Chuto haplotype, whereas European haplotypes clustered into a new basal phylogroup (Figure 1).

## Conclusion

We have established the presence of *Orientia* DNA in spleens of rodents from Thailand, as was expected, but also in rodents collected from France and Senegal. In Asia, scrub typhus is considered as a seriously neglected life-threatening disease despite the fact that this ancient disease has been recognized within this region for many years. Our findings, together with those from other recent studies [4,5] suggest that in locales outside of Asia where the disease is not on the public health service radar, surveillance needs to be improved.

## Ethical approval

Animals have been treated in accordance with the guidelines of the European Union legislation (Directive 86/609/EEC). The CBGP laboratory has received the approval (no. B 34–169–1) from the regional Head of Veterinary Service (Hérault, France), for the sampling and killing of rodents and the harvesting of their tissues. Dr Cosson has personally received the agreement “certificate d'autorisation d’expérimenter sur animaux vivants” (i.e. “certificate of authorization to experiment on live animals”) (no. C34–105) by the French administration.
